# Topical Anti-Inflammatory Activity of Essential Oils of *Alpinia calcarata* Rosc., Its Main Constituents, and Possible Mechanism of Action

**DOI:** 10.1155/2020/2035671

**Published:** 2020-04-27

**Authors:** Madhuvanthi Chandrakanthan, Shiroma M. Handunnetti, Galbada Sirimal Arachchige Premakumara, Selvaluxmy Kathirgamanathar

**Affiliations:** ^1^Industrial Technology Institute, Colombo-07, Sri Lanka; ^2^Institute of Biochemistry, Molecular Biology and Biotechnology, University of Colombo, Colombo-03, Sri Lanka; ^3^Faculty of Nursing, University of Colombo, Sri Jayewardenepura, Thalapathpitiya, Nugegoda, Sri Lanka

## Abstract

This study aimed at investigating the anti-inflammatory potential of essential oil from rhizome and leaf of *Alpinia calcarata* Rosc. (ACEO) with the focus of its topical anti-inflammatory activity along with its dominant compounds 1,8-cineole and *α*-terpineol using mouse ear edema model. ACEOs were analyzed by GC-MS. The anti-inflammatory activity was determined by studying the inhibition of overproduction of proinflammatory mediators—nitric oxide, reactive oxygen species, prostaglandins, cyclooxygenases, and cytokines induced by lipopolysaccharides in murine macrophages. Topical anti-inflammatory and antinociceptive activity was studied by 12-O-tetradecanoylphorbol-13-acetate (TPA) induced skin inflammation and formalin-induced pain model in mice, respectively. Rhizome oil has 1,8-cineole (31.08%), *α*-terpineol (10.31%), and fenchyl acetate (10.73%) as major compounds whereas the ACEO from leaves has 1,8-cineole (38.45%), *a*-terpineol (11.62%), and camphor (10%). ACEOs reduced the production of inflammatory mediators *in vitro* in a concentration-dependent manner. Further, ACEO and its major compounds reduced ear thickness, weight, myeloperoxidase, and cytokines significantly (*p* < 0.01) in mouse ear. Dose-dependent reduction in flinching and licking in both the phases of pain sensation concludes the topical analgesic effect. Our findings suggest the potency of topical use of ACEOs for inflammatory disease conditions.

## 1. Introduction

Inflammation is a defending process exhibited by organisms against noxious stimuli, marked by an intensified blood influx to the affected tissue resulting in pain, heat, swelling, redness, and loss of function of the affected part [1, 2]. Referred commonly as “double-edged sword,” it aids in the elimination of pathogens, whereas uncontrolled inflammation could lead to tissue injury and neoplastic transformation [3]. Further, inflammation-related acute and chronic diseases are accompanied by pain which subjugates the quality of life and overall productivity [4]. Macrophages, the remarkably plastic cells of the immune system, get activated in the inflammatory process, thereby producing proinflammatory mediators such as nitric oxide (NO), PGE2 (prostaglandin E2), COX 1 and 2 (cyclooxygenase 1 and 2), reactive oxygen species (ROS), and cytokines [5]. Skin acts as the primary interface between the body and the external environment and provides the first line of defence against disease-causing pathogens and traumatic injury [6]. In addition, as a physical barrier [7], the skin has many active immune defence mechanisms. A breach in the immunological balance can head to acute and chronic inflammatory skin diseases such as psoriasis and allergic contact dermatitis [8]. In this condition, topical treatments of skin diseases have combined benefits that include simplicity in application, escaping of hepatic first-pass metabolism, attaining maximum efficacy with less drug dosage, easy termination of drug if needed, site-specific drug delivery, high adherence, and risks associated with oral or intravenous administration [9, 10]. Further, topical anti-inflammatory agents can inhibit the variety of factors and mediators of inflammation such as expression of cytokines, growth factors, adhesion molecules, nuclear factor-*κ*B (NF-*κ*B), nitric oxide, and prostanoids [11]. TPA-induced skin inflammation is a widely used model to study the anti-inflammatory effects of natural and chemically synthesized drugs. High levels of inflammatory cytokines and reactive oxygen species are proposed to contribute to the pathophysiological mechanisms associated with TPA-induced cutaneous inflammation [12]. Conventional nonsteroidal anti-inflammatory drugs (NSAIDs) ameliorate the inflammation by suppressing the mediators and also act as local analgesics [13]. However, evidences suggest that the long-term use of NSAIDs may induce gastrointestinal ulcers, bleeding, and renal disorders leading to the exploration of less or noninvasive therapeutics which could aid in the treatment strategy of the acute and chronic inflammatory diseases [14].

*Alpinia calcarata* Rosc. (Zingiberaceae) is a widespread perennial plant throughout the tropical and subtropical Asian countries including Sri Lanka, India, Bangladesh, Thailand, and Malaysia [15, 16]. Rhizomes have been commonly used in Sri Lankan and Indian traditional medicine to treat chronic inflammatory diseases such as rheumatism and asthma [17]. Studies on the hot water, ethanolic extract, and oil extract of rhizome exhibited potent anti-inflammatory activity in carrageenan-induced mice models [18–20]. Previous findings reported that extracts of AC had antimicrobial, antifungal, antihelminthic, antinociceptive, antioxidant, aphrodisiac, gastroprotective, and antidiabetic properties [21]. Earlier, researchers have reported the chemical composition of ACEO grown in Sri Lanka to be rich in oxygenated monoterpenes with 1,8-cineole as the major constituent of rhizome and leaf EOs [22]. But this study lacks to give the detailed profile of volatile constituents from flowering AC grown in Sri Lanka. Similar supporting reports have been documented with ACEOs from germplasms in South India [23–26]. Further, the main constituents 1,8-cineole (CIN) and *α*-terpineol (TPN) have been known to act as anti-inflammatory agents *in vivo* [27, 28]; its topical anti-inflammatory effect and mechanism of action for skin diseases such as atopic dermatitis were never reported. Taking this into account, we postulated that ACEO which is rich in monoterpenes like 1,8-cineole and *α*-terpineol could be effective in preventing TPA-induced acute skin inflammation in mice and inhibit inflammatory mediators *in vitro*. With the existing knowledge from literatures, this is the first study that reports the detailed profile of volatile constituents, topical anti-inflammatory activity, and *in vitro* mechanism of action of AC. To test this possibility, we have studied the effects of ACEO and main constituents on the TPA-induced cutaneous inflammation. In order to determine ACEOs mechanism *in vitro*, RAW 264.7 cells induced with LPS have been used to measure the NO, ROS, cytokines, COX, and PGE2. In addition, the cytotoxicity of the biologically active ACEOs was also evaluated on macrophages, intestinal epithelial cells, human hepatocytes, and keratinocytes in order to assess the effect if targeted for oral or topical application and further gauge the therapeutic edge over the existing drugs.

## 2. Materials and Methods

### 2.1. Plant Material

Whole plants of *A*. *calcarata* were collected from the Western province of Sri Lanka in 2015 during the flowering season. The plants were authenticated by N. P. T. Gunawardena, and voucher specimens were deposited at National Herbarium, Peradeniya, Sri Lanka (Voucher Specimen Number: 6/01/H/03).

### 2.2. Chemicals

Luminol (3-aminophthalhydrazide), HBSS (Hank's balanced salt solution), zymosan A (*Saccharomyces cerevisiae* origin), DMSO (dimethylsulphoxide), aspirin (acetylsalicylic acid), indomethacin, diclofenac, dexamethasone, NMMA (N^G^-methyl-L-arginine acetate salt), PTIO (2-phenyl-4,4,5,5-tetramethylimidazoline-1-oxyl 3-oxide), NADH (*β*-nicotinamide adenine dinucleotide), 12-O-tetradecanoylphorbol-13-acetate (TPA), PMS (phenazine methosulfate), formaldehyde (37%), LPS (lipopolysaccharides (*Escherichia coli* origin)), Dulbecco's modified Eagle's medium (DMEM), fetal bovine serum (FBS), 3-(4,5-dimethyl-2-thiazolyl)-2,5-diphenyl tetrazolium bromide (MTT), NBT (nitrotetrazolium blue chloride), H_2_O_2_ (hydrogen peroxide solution)_,_ sulfanilamide, *N*-(1-naphthyl)ethylenediamine dihydrochloride (NED. 2 HCl), sodium nitroprusside dihydrate (SNP), 1,8-cineole, and *α*-terpineol were procured from Sigma (USA). Murine macrophages (RAW 264.7), human hepatocellular carcinoma (HepG2), human keratinocytes (HaCaT), and rat intestinal epithelial cells (IEC-6) were procured from National Centre for Cell Sciences, Pune, India. TNF-*α*, IL-1*β*, and IL-6 assay kits were purchased from Becton Dickinson (BD), USA. COX activity assay kit was purchased from Abcam (USA).

### 2.3. Animals

Eight-week-old male and female Swiss albino mice (18–22 g) from a colony maintained at the in vivo Testing Facility, Central Institute of Medicinal and Aromatic Plants, India, were used for the experiments. The mice were housed under standardized conditions (25 ± 2°C), 12 h light/12 h dark cycle, and fed with animal pellets and water ad libitum. The protocol (CIMAP/IAEC 2016-19/05) was duly approved by the Institutional Animal Ethics Committee (IAEC).

### 2.4. Extraction of ACEOs

The whole plant was washed, and the rhizome and leaves were chopped separately. Each part (450 g) was separately hydrodistilled for 4 h using 500 mL distilled water in a Clevenger-type apparatus to obtain the essential oils. After decanting, oil samples were dried with anhydrous Na_2_SO_4_ and stored at 4°C prior to analysis. The percentage yield of oil was calculated as the ratio of weight of the oil to the weight of the fresh plant part separately.

### 2.5. Gas Chromatography-Mass Spectrometry (GC/MS) Analysis of ACEOs

The ACEOs from rhizome and leaf were analyzed by Thermo Scientific™ TRACE™ 1300 Series GC operated with a split mode injector, Thermo Scientific AI/AS 1310 Series autosampler, and Thermo Scientific™ ISQ™ Series GC-Single Quadrupole MS. The following were the specifications used for analysis: column: TG Wax MS (acid-deactivated polyethylene glycol) 30 m, 0.25 mm i.d., 0.25 *μ*m film thickness (Thermo Scientific, USA); temperature program: from 60°C to 150°C at 3°C/min and from 150°C to 240°C at 7°C/min; injector temperature: 240°C; injection volume: 1.0 *μ*L; inlet pressure: 86.3 kPa; carrier gas: He; flow rate: 1.000 mL/min; injection mode: split (50 : 1); mass interface temp.: 250°C; MS mode: EI; detector voltage: 70 eV; mass range: 40–450; and interval: 0.2 sec. Data handling was made through Xcalibur software. The relative amount of individual components of the total oil is expressed as percentage peak area relative to total peak area. Qualitative identification of the different constituents was performed by comparison of their relative retention times and mass spectra with those of authentic reference compounds, or by retention indices (RI) and mass spectra. Compound identification was done by comparing the National Institute of Standards and Technology (NIST 2014) library data of the mass spectra peaks with those reported in the literature.

### 2.6. Measurement of Cytotoxicity and Proliferative Index (PI)

The cytotoxic effects of the ACEOs were carried out on RAW 264.7, HepG2, IEC-6, and HaCaT cells through MTT assay [29] for cytotoxicity and proliferative index by the same procedure. A total of 5 × 10^4^ cells/well were seeded on to 96-well plates, and upon reaching the desired confluence, the cells were incubated with three different concentrations of ACEOs (0.5, 5, and 50 *μ*g/mL) in complete culture medium, not exceeding 0.5% DMSO in content for 24 h at 37°C and 5% CO_2_. Incubation of cells with culture medium containing DMSO at a final concentration of 0.5% (*v*/*v*) was used as a negative control. The absorbance of developed formazan in the treated cells was quantified at 595 nm in a microplate reader against the DMSO (SpectraMax 384 Plus, Molecular Devices, USA). The proliferative index was carried out with a similar protocol, except the element that the cells were cultured in serum-free medium at the concentrations of 0.5, 5, and 50 *μ*g/mL. The PI was calculated as percentage inhibition of proliferation by calculating the absorbance difference between untreated cells and treated cells.

### 2.7. In Vitro Anti-Inflammatory Assays

#### 2.7.1. Nitric Oxide and iNOS Production Inhibition and Nitrite Scavenging Potential

RAW 264.7 cells were treated with LPS (1 *μ*g/mL) in the presence of various concentrations of ACEOs 0.5, 5, and 50 *μ*g/mL and compounds (0.32–1.25 *μ*g/mL) for 4 h followed by removal of culture supernatant containing ACEO and added LPS containing culture media. After 20 h, the concentration of nitrite, the stable product of NO, was quantified in the culture supernatant by Griess reagents (1% sulfanilamide and 0.1% NED.2HCl) as described previously [30]. The amount of nitrite in the sample was calculated from a sodium nitrite standard curve, and the absorbance was measured by Synergy HTX multimode reader (BioTek Instruments, United States). In an attempt to determine the involvement of iNOS in NO production inhibition by ACEOs, RAW 264.7 cells were induced by LPS for 12 h prior to treatment with ACEOs for 24 h. *In vitro* stimulation of cells to an inflammatory state prior to treatment with drug enables the synthesis of intracellular iNOS and accumulation of high levels with corresponding enhanced synthesis and secretion of NO [31]. L-NMMA was used as a specific inhibitor of iNOS enzyme activity (positive control). The supernatants were removed and assayed for nitrite using the Griess assay as described above. In a separate experiment, the free radical nitrite scavenging ability of ACEOs was estimated by generating a NO production system with SNP (10 mM) and phosphate buffer (pH 7.4), followed by the addition of Griess reagent, and the absorbance was measured. PTIO, a synthetic nitrite scavenger, was used as a positive control.

#### 2.7.2. Measurement of Intracellular ROS Production

The inhibition of intracellular ROS production by ACEOs was quantified through chemiluminescence as described by Koko et al. [32]. Briefly, RAW 264.7 cells (1 × 10^5^ cells/well) were suspended in HBSS with Ca^2+^ and Mg^2+^ (pH 7.4) and treated with varying concentrations of ACEO (1.56–50 *μ*g/mL) followed by incubation at 37°C for 30 min in the thermostated chamber of the BioTek SynergyTM multimode reader. The production of ROS was initiated by the addition of opsonized zymosan A followed by 25 *μ*L luminol, and the volume was adjusted to 200 *μ*L with HBSS^++^. The results were monitored as relative luminescence units (RLUs) with peak and total integral values set with repeated scans at 30 s intervals for 1 h.

#### 2.7.3. Superoxide Radical Scavenging Activity

Superoxide anion scavenging activity was measured in sodium phosphate buffer (100 mM, pH = 7.4) containing NBT solution (150 *μ*M), NADH solution (468 *μ*M), and different concentrations (1.56–50 *μg*/mL) of ACEOs. The reaction was started with the addition of PMS (60 *μ*M) to the mixture followed by incubation at 25°C for 5 min and measurement of the absorbance at 560 nm. Compared with the optical density (OD) with no test sample added, the reduction of the absorbance was quantified as the superoxide scavenging activity.

#### 2.7.4. Measurement of Cytokine Production

Secreted cytokine levels were evaluated by incubating RAW 264.7 cells induced with 1 *µ*g/mL of LPS and treated with ACEOs at 0.5, 5, and 50 *μg*/mL for 4 h. The modulatory activity of ACEO on the LPS-induced production of TNF-*α*, IL-1*β*, and IL-6 was quantified through EIA (BD, USA) using the culture supernatants of RAW 264.7 cells collected after 24 h of incubation. Dexamethasone at 10 *μ*M was used as the positive control.

#### 2.7.5. Measurement of PGE2 Levels

RAW 264.7 cells were induced with LPS 1 *μ*g/mL together with ACEOs at 0.5, 5, and 50 *μ*g/mL for 4 h. After 4 h, ACEO-containing media was removed and replaced with LPS-containing media and incubated for 24 h. After 24 h, culture supernatant was collected for ELISA quantification of PGE2 using PGE2 assay kits (ParameterTM; R&D Systems, MN, USA). The PGE2 standard and the RD5-39 in the kits were used to construct a standard curve. Culture medium (100 *μ*L) was mixed with 50 *μ*L of primary antibody solution and PGE2 conjugate and incubated for 2 h at room temperature with continuous shaking. Wells were then washed using 400 *μ*L of washing buffer followed by addition of colour reagent (200 *μ*L), and 30 min later, stop solution (50 *μ*L) was added. Absorbance was measured at 450/570 nm using a BioTek Synergy HTX multimode reader. Indomethacin was used as the positive control.

#### 2.7.6. In Vitro COX Inhibition Assay

The efficacy of ACEOs to inhibit ovine COX-1 and COX-2 was determined using an enzyme immunoassay (EIA) kit (catalog no. 560101; Cayman Chemical Co., Ann Arbor, MI, USA). COX catalyzes the first step in the biosynthesis of arachidonic acid (AA) to PGH_2_. PGF_2*α*_, produced from PGH_2_ by reduction with stannous chloride, was measured by EIA (ACE™ competitive EIA, Cayman Chemical, Ann Arbor, MI, USA). Briefly, to a series of supplied reaction buffer solutions (160 *μ*L 0.1 M Tris-HCl (pH 8.0) containing 5 mM EDTA and 2 mM phenol) with either COX-1 or COX-2 (10 *μ*L) enzyme in the presence of heme (10 *μ*L), 10 *μ*L of various concentrations of ACEO (0.5, 5, and 50 *μ*g/mL) were added. These solutions were incubated for 5 min at 37°C, and subsequently, 10 *μ*L AA solution (100 *μ*M) was added. Further, it was incubated for 2 min at 37°C. The COX reaction was stopped by the addition of 30 *μ*L of stannous chloride and incubated for 5 min at room temperature. Prostaglandins, PGF_2*α*_, produced were quantified by ELISA. This assay is based on the competition between PGs and a PG-acetylcholinesterase conjugate (PG tracer) for a limited amount of PG antiserum. Standard and samples were mixed with PG screening AChe tracer and PG antiserum and was incubated for 18 h at room temperature. After incubation, the plate was washed to remove any unbound reagent, followed by addition of Ellman's reagent (200 *µ*L), and the mixture was incubated for 60 min at room temperature (until the absorbance of Bowell is in the range of 0.3–1.0 A.U.). The product of this enzymatic reaction develops a yellow colour that absorbs at 412 nm. The intensity of this colour is proportional to the amount of PG tracer bound to the well, which is inversely proportional to the amount of PGs present in the sample. Percent inhibition was calculated by the comparison of the compounds treated to the various control incubations.

#### 2.7.7. Assay for Membrane Stabilization

Fresh whole human blood (2 mL) was collected from a healthy volunteer and was washed three times with normal saline and constituted as 40% *v*/*v* suspension with normal saline as described by Sadique et al. [33]. The reaction mixture consisted of ACEOs (1.56–50 *μ*g/mL) or dexamethasone (10 *μ*M) and 20 *μ*L of 40% RBCs suspension, considering only RBCs as controls. The reaction mixture (in triplicate) after incubation in a water bath (54°C for 25 min) was centrifuged at 2500 rpm for 5 min, and the absorbance of the supernatants was taken at 560 nm. Percent membrane stabilization activity was derived mathematically. The ethical approval for collection of blood from human donors was obtained from Research Ethics Committee, Institute of Biochemistry, Molecular Biology and Biotechnology, University of Colombo, Sri Lanka.

### 2.8. Topical Anti-Inflammatory Activity

#### 2.8.1. TPA-Induced Skin Inflammation

Acute inflammation in the ear pinna was induced by instilling 20 *μ*L of TPA dissolved in acetone (2.5 *μ*g/ear). Mice were divided into 9 groups with 6 animals per group. TPA (2.5 *μ*g/ear) dissolved in 20 *μ*L of acetone was applied to the inner and outer surfaces of mouse ears with the aid of a micropipette [34]. Treatments, ACEOs (5.0, 1.0, and 0.2%), CIN (2.5, 0.5, and 0.1%), and TPN (10, 2, and 0.4%) or indomethacin (0.5 mg/ear) was applied after the TPA induction. Four h later, the thickness of the ear was measured using a digital screw gauge. Six h after the treatment, the animals were euthanized for the collection of tissue. Ear biopsies (5 mm diameter punches) were weighed, homogenized in PBS (pH 7.5) with 1 mM EDTA, and centrifuged (10,000 ×*g* for 15 min) for the collection of supernatant which were used for the quantification of various cytokines.

#### 2.8.2. Histopathological Analysis of Mouse Ear Tissue

For the assessment of skin inflammation, biopsies from control and treated ears of mice in each treatment group were collected and fixed in 4% formaldehyde (0.1 M phosphate buffer, pH 7.4). Subsequently, the tissues were dehydrated, blocked in paraffin, and serially sliced at a thickness of 5.0 *μ*M using a microtome (Leica Microsystems, USA). The sections were stained with hematoxylin-eosin (H & E), and a representative section from each group of animals was selected to show the histopathological changes. The selected sections were analyzed by light microscopy (Leica Microsystems, USA), and the images were captured at 10x and 40x magnifications. The measurements of ear thickness and epidermal thickness (*μ*m) were acquired by the Image Processing software of Leica Microsystems.

#### 2.8.3. Assay for Myeloperoxidase Enzyme Activity in Ear Tissue

MPO activity was quantified according to the method proposed by Pulli et al. [35]. Briefly, the homogenate was added with a mixture containing 80 mM·PBS (pH 5.4), 0.22 M·PBS (pH 5.4), and 0.017% hydrogen peroxide. The reaction was initiated by the addition of 20 *μ*L of 18.4 mM TMB in dimethylsulphoxide. The plate was incubated at 37°C for 4 min followed by the addition of 1.46 M sodium acetate (pH 3.0) to stop the reaction. The absorbance at 620 nm was measured using a plate reader to determine the enzyme activity. MPO activity was expressed as absorbance of TPA-treated ear tissue homogenate ÷ absorbance of ACEO-treated ear tissue homogenate.

#### 2.8.4. Formalin-Induced Nociception and Edema in Mice

The procedure described by Lee and Jeong [36] was followed. Briefly, nociception was induced by injecting 20 *μ*L of 2.5% formalin in 0.9% saline in the subplantar region of the right hind paw. Mice (*n* = 6, per group) were pretreated topically with ACEOs (5%, 1%, and 0.25%), CIN (2.5, 0.5, and 0.1%), TPN (10, 2, and 0.4%), 1% diclofenac cream, and vehicle for 1 h prior to injecting formalin. These mice were individually placed in a transparent glass chamber for observation. The amount of time spent licking and flinching of the injected paw was indicative of pain. The number of flinches and lickings after injection of formalin was counted during 0 to 5 min (early phase) and 20 to 30 min (late phase). Antinociception was considered as a statistically substantial reduction in the time spent in licking and flinching of the injected paw in comparison with the control group. Edema was estimated by measuring the paw volume before and after 4 h of formalin injection using plethysmometer (IITC, Life Scientific Instruments, Woodland Hills, CA, USA). The percentage reduction in paw volume by ACEOs was calculated in comparison with the formalin-treated group.

### 2.9. Statistical Analyses

The raw data were analyzed by *t*-tests and one-way ANOVA (analysis of variance) followed by Tukey's test and Dunnett's comparison test, where applicable using GraphPad Prism version 5.0 (GraphPad Prism Software Inc., San Diego, CA, USA). Differences were considered to be statistically significant when *p* < 0.05.

## 3. Results and Discussion

### 3.1. Analysis of Essential Oil

**Figure 1:**
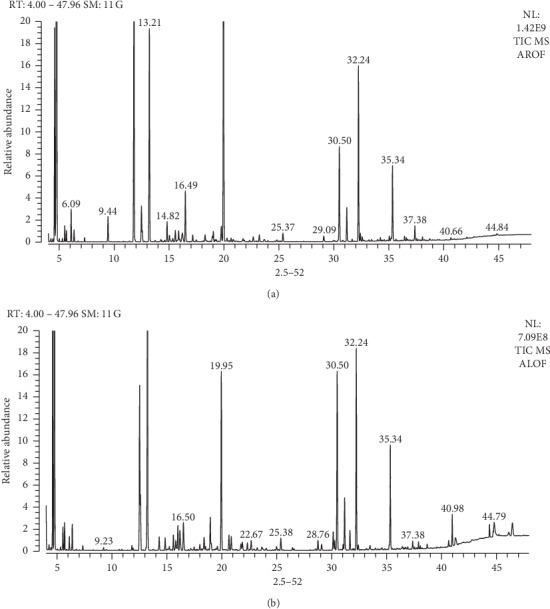
GC-MS profile of rhizome (a) and leaf (b) essential oils of AC.

The yield of essential oil from rhizome and leaf was 9 and 12 mL/kg, respectively. Results are the summary of three batches of analysis. Essential oils are complex mixtures consisting of various compounds. Each of these compounds contributes to the beneficial or adverse activity of the essential oil. For this reason, it is necessary to elucidate the complete composition of an essential oil when investigating the viability of a specific application [37]. GC-MS equipped with a capillary column is the most popular technique used to analyze the chemical ingredients of an essential oil. The list of compounds in ACEO from rhizome and leaf is presented in Table 1 and Figures 1(a) and 1(b). Previous study on the volatile oils of AC grown in Sri Lanka reports 3.60% and 0.42% of oil yield from dry rhizome and fresh leaves, respectively [22]. Earlier reports of rhizome EO from germplasms collected from India showed variation ranging from 0.29% to 0.96% in rhizome and 0.26% to 0.69% in aerial leafy shoots on a dry weight basis [38]. All the previous studies were done on either dry or fresh rhizome and aerial parts, and the information on the harvest stage was not mentioned. In our study, oils were collected from flowering plants. Fresh rhizome constituted mainly of oxygenated monoterpenes (71.28%), followed by monoterpenes (9.64%), sesquiterpenes (5.4%), and oxygenated sesquiterpenes (0.15%). The major compounds identified were 1,8-cineole (38.45%), *α*-terpineol (11.62%), and fenchyl acetate (10.73%). The compounds in leaf oil were also occupied by the same order, and the major compounds were 1,8-cineole (31.08%), *α*-terpineol (11.62%), and camphor (10%). The observations made in the current study gave a detailed profile of the essential oils from rhizome and leaf and also support the previous reports from different origins on the EO content of AC marked with 1,8-cineole as the major component independent of the flowering or condition of the plants [39]. The most important chemical feature of the oils obtained from flowering stages of AC was the presence of a higher percentage of oxygenated monoterpenes, mainly 1,8-cineole and fenchyl acetate which were of industrial interest in terms of bioactivity.

### 3.2. Cytotoxicity and Proliferative Inhibition Effects of ACEOs

**Figure 2:**
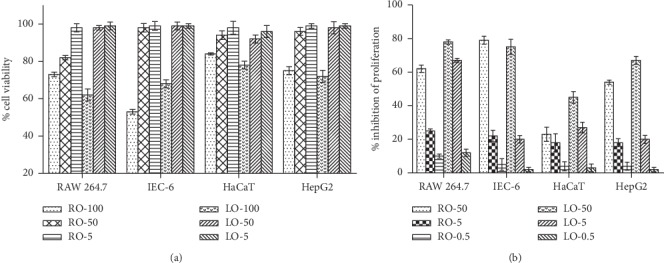
Cytotoxic effect of ACEOs (100, 50, and 5 *μ*g/mL) on RAW 264.7, HaCaT, HepG2, and IEC-6 cell lines. Cell viability (a) and proliferative index (b) of ACEOs (50, 5, and 0.5 *μ*g/mL) on RAW 264.7, HaCaT, HepG2, and IEC-6 cell lines. RO: rhizome essential oil; LO: leaf essential oil.

Figures 2(a) and 2(b) show the effect of ACEOs on RAW 264.7, HaCaT, HepG2, and IEC-6 cell viability and proliferative ability. Treatment with 100 *μ*g/mL caused a reduction in cell viability in all the above cell lines. Therefore, ACEOs were considered at 50 *μ*g/mL and below for further assays. Considering that intestinal epithelial cells and keratinocytes are the first point of contact when drugs are used orally or applied topically and virtually, all NSAIDs that have been used extensively are linked to at least rare cases of clinically apparent drug-induced liver injury [40]. Hence, the need to analyze the cytotoxicity becomes a necessity to identify safe and bioactive concentrations to mammalian cells for oral and topical curative applications. At 100 *μ*g/mL, RAW 264.7, IEC-6, and HepG2 cells have less than 80% viability (Figure 2(a)). However, HaCaT cells were not affected at 100 *μ*g/mL, which gives an interesting niche, suggesting a potential safe application of these ACEOs in topical therapeutics. Further, the proliferative inhibition efficacy of ACEOs was tested in order to explore the efficacy for prolonged inflammatory diseases, since chronic inflammation leads to neoplastic transformation of cells with the aid of proinflammatory markers [41]. Recent evidences on the TNF-*α* and IL-6-induced tumour progression of inflammatory cells [42] urge the search for anti-inflammatory drugs with tumour inhibition potential. In the present study, ACEOs exhibited inhibition of proliferation in a concentration-dependent manner in all the cell lines tested (Figure 2(b)). Among the 4 cell lines tested, HaCaT cells are the least affected with 23 ± 4.23 and 45 ± 3.45 percentage inhibition of proliferation at 50 *μ*g/mL for RO and LO, respectively.

### 3.3. Inhibition of NO Release, Indirect iNOS Activation, and Free Radical Nitrite Scavenging of ACEOs

**Figure 3:**
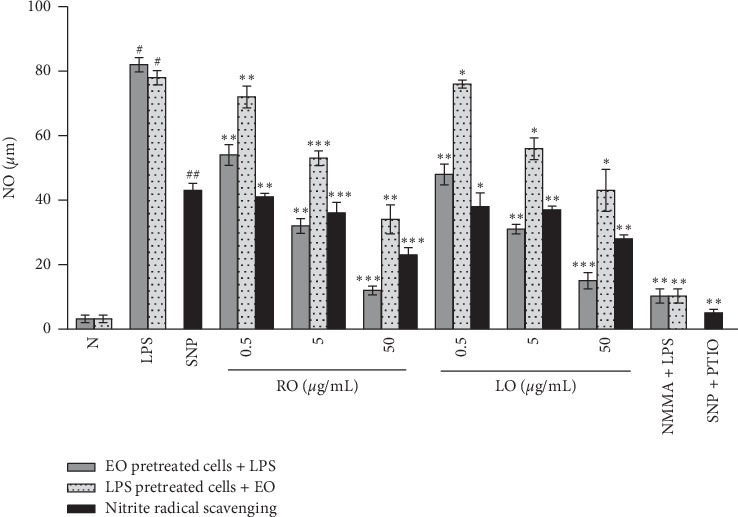
Effect of EOs of AC on nitric oxide (NO) inhibition. Effect of ACEOs on NO production, iNOS activation, and nitrite scavenging at 0.5, 5, and 50 *μ*g/mL. N: cells not treated with LPS or drug; LPS: cells treated with LPS (1 *μ*g/mL); SNP: free radical NO generation by SNP; RO: rhizome essential oil; LO: leaf essential oil; L-NMMA + LPS: cells treated with 250 *μ*M L-NMMA and LPS; SNP + PTIO: free radical NO generation by SNP with nitrite scavenger PTIO. The data are represented as mean ± SEM (*n* = 6). Symbols “^*∗*^,” “^*∗∗*^,” and “^*∗∗∗*^” represent a significant difference *p* < 0.05, *p* < 0.01, and *p* < 0.001, between LPS+ and EO-treated cells. Symbols “^#^” and “^##^” represent a significant difference *p* < 0.05 between normal and LPS + cells, buffer, and SNP generated NO.

ACEOs were tested for anti-inflammatory activity by the effect of them in LPS-induced NO production in murine macrophages.  Figure 3 shows the concentration-dependent response of ACEOs towards the production of NO, indirect iNOS, and nitrite scavenging. LPS-induced RAW 264.7 cells produce large amounts of NO and the stable product, nitrite, which was measured by the colourimetric Griess assay. ACEOs exhibited significant inhibition of NO production at all concentrations tested in a concentration-dependent manner. EO from rhizome showed maximum inhibition of 85% at 50 *μ*g/mL, whereas the leaf oil showed 81% inhibition. L-NMMA was used as a positive control at a concentration of 250 *μ*M, and it exhibited NO inhibition of 87% when RAW 264.7 cells were treated with LPS for 24 h. NO is a short-lived signaling molecule that plays an important role as an immunoregulatory mediator [43]. High NO levels cause a variety of pathophysiological processes including inflammation and carcinogenesis [44]. In addition, regulation of the iNOS-mediated release of NO from macrophages is considered as one of the strategies to develop therapeutics against various inflammatory diseases such as rheumatoid arthritis [45]. To investigate the involvement of iNOS in the mechanism of inhibition of NO production by ACEOs, RAW cells were treated with LPS for 12 h prior to the treatment with ACEOs, which showed a reduced NO production at the rate of 57.5% and 46.25% for rhizome and leaf, respectively, at 50 *μ*g/mL. It also showed a concentration-dependent inhibition that further confirms the iNOS enzyme-mediated inhibition of NO production which facilitates the inhibition of iNOS gene expression at the genome transcriptional level. Further, ACEOs were tested for the scavenging activity of nitrite generated by SNP mediation that exhibited the moderate scavenging activity from rhizome (46.5%) and leaf (34.8%). This adds to the effect of ACEOs on NO inhibition which is by the inhibition of enzyme (iNOS) and not by mere scavenging activity.

### 3.4. Inhibition of Intracellular ROS Production and Superoxide Scavenging Activity by ACEOs

ACEOs were studied for the inhibition of oxidative burst induced by opsonized zymosan (OPZ) by a luminol-dependent chemiluminescence assay. Induction by OPZ caused the activation of NADPH oxidative complex which results in the production of ROS and this is mainly expressed in phagocytic cells of the immune system [46]. Prolonged local inflammatory reaction triggers the production of excessive amounts of ROS, and since macrophages have the highest burst capacity among the antigen-presenting cells, this harmful massive burst further triggers the immune activation of proinflammatory cytokine synthesis and results in tissue damage [47]. In the present study, the potent inhibitory activity was observed for the leaf EO (87% inhibition) and both the ACEOs exhibited a concentration-dependent inhibition of ROS production, as shown in Table 2. OPZ triggers both interferon-*γ* and complement receptor activation producing superoxide anions that are spontaneously converted to halide ions by the oxidative enzymes [48] aggravating the oxidative burst. Luminol has its ability to enter the cell, which reacts with intracellular HOCl^−^ exhibiting the ability of ACEOs in the inhibition of oxidative burst. In a separate experiment, superoxide anions were generated by incubating PMS with NADH, and the free radical O_2_^−^ scavenging ability was studied in a quest to identify the extracellular superoxide scavenging activity of ACEOs. Interesting observation was obtained with the less inhibition pattern of 10–13% for the higher concentrations of ACEOs tested (Table 2). Earlier reports also indicated the essential oils as moderate scavengers of free radicals [49].

### 3.5. Inhibition of Cytokine Secretion by ACEOs

**Figure 4:**
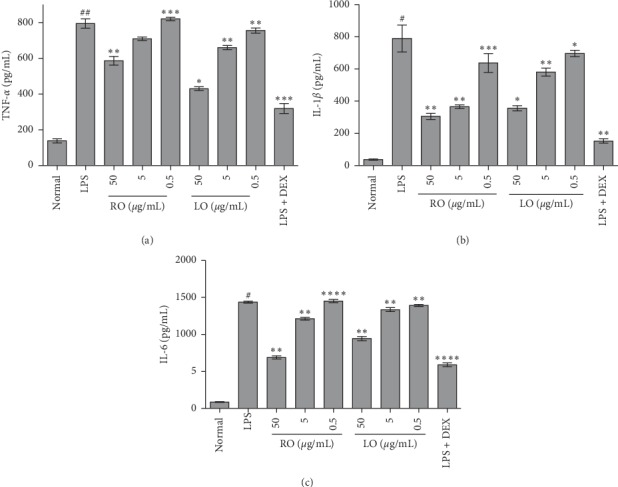
Effects of ACEOs on LPS-induced cytokine production in murine macrophages. TNF-*α* levels in supernatant (a), IL-1*β* levels in supernatant, (b) and IL-6 levels in supernatant (c). N: normal; LPS: LPS-treated cells; RO: rhizome essential oil; LO: leaf essential oil; LPS + DEX: LPS along with dexamethasone at 10 *μ*M. The bars represent the mean ± SEM (*n* = 6). Symbols “^∗^,” “^∗∗^,” and “^∗∗∗^” represent a significant difference *p* < 0.05, *p* < 0.01, and *p* < 0.001, between LPS+ and EO-treated cells. Symbol “^#^” represents a significant difference *p* < 0.05 between normal and LPS + cells.

Figures 4(a)–4(c) show the amount of cytokines quantified in the culture medium of RAW 264.7 cells treated with several concentrations of ACEOs prior to LPS induction for 3 h and further incubated for 24 h with LPS. Pretreatment of RAW 264.7 cells with ACEOs markedly decreased the production of TNF-*α*, IL-1*β*, and IL-6 when compared to the LPS-treated cells. Macrophages react to the LPS by recognizing the threat using the Toll-like receptor complex (TLR-4) which results in the intensified production of NO, ROS, and cytokines [50]. Intensified production of cytokines in the activated macrophages of the inflammatory process results in the tissue damage as seen in chronic inflammatory diseases [51]. Treatment with ACEOs significantly inhibited the production of cytokines by LPS (*p* < 0.05). Interestingly, ACEOs also decreased the proliferation of macrophages along with the inhibition of cytokine induction which makes it an effectual lead for the preparation of formulations for chronic disorders [52].

### 3.6. Effect of ACEOs on PGE2 Production

**Figure 5:**
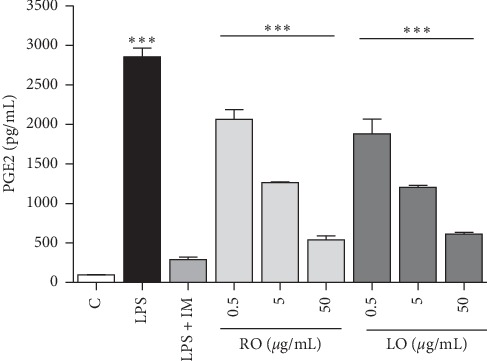
Effect of ACEOs on PGE2 production. C: normal control; LPS: LPS-treated cells; RO: rhizome essential oil; LO: leaf essential oil; LPS + IM: LPS along with indomethacin at 10 *μ*g/mL. The bars represent the mean ± SEM (*n* = 6). Symbol “^*∗∗∗*^” represents a significant difference *p* < 0.001, between control and LPS-treated cells, and comparing LPS+ and EO-treated cells.

PGE2 is an eicosanoid lipid mediator produced when arachidonic acid is released from the plasma membrane by phospholipases and metabolized by two cyclooxygenases (COX 1 and COX 2) and three specific isomerases. NSAIDs act on COX enzymes, thus reducing the generation of PGE2. Thus, downstream pathways in the COX enzymes are responsible for response of PGE2 and are more specific targets in the treatment of inflammation and pain [53]. The effects of ACEO on the LPS-induced release of PGE2 from RAW 264.7 cells were studied. As PGE2 is one of the most important inflammatory mediators, the cells were pretreated with ACEOs for 2 h followed by incubation with 1 *μ*g/mL of LPS. After 24 h of LPS treatment, the PGE2 contents in the culture medium were detected. The LPS-induced PGE2 secretion level was inhibited by treatment with the ACEOs at all the concentrations examined, and the maximum inhibition was observed at a concentration of 50 *μ*g/mL for both oils (Figure 5). Indomethacin (IMN) was used as a positive control. Diverse studies have proven that the expression of COX-2 is largely determined by transcriptional activation [54, 55]. NF-*κ*B, which is a mammalian transcription factor that regulates several genes and important in immunity and inflammation, can be triggered by LPS and other proinflammatory cytokines. In macrophages, NF-*κ*B binds to COX-2 promoter and plays a role in LPS-mediated induction of COX-2. In addition, binding of CCAAT-enhancer-binding proteins (C/EBPs), c-AMP response element binding proteins (CREBs), and c-Jun to the COX-2 promoter ameliorates its transcriptional activation [56]. The present study is confined to the understanding of *in vitro* enzyme inhibitory activity of COX-1 and COX-2 and the production of PGE2; thence, it may be significant to understand the effect of ACEOs at the transcriptional activation level involving the NF-*κ*B, C/EBP, CREB, and c-Jun proteins.

### 3.7. In Vitro COX Enzyme Inhibition by ACEOs

COX-1 and COX-2 catalyze the biosynthesis of PGH2 from the AA substrate. The COX-1 inhibition results in certain unsuitable side effects, whereas COX-2 inhibition provides therapeutic effects in pain, inflammation, cancer, Alzheimer's disease, and Parkinson disease [57]. Therefore, the present study aimed at examining the COX-1 and COX-2 inhibitory activity of ACEO on purified enzymes as a mechanism of anti-inflammatory action. The oils showed inhibitory effects on COX-1 and COX-2 in a concentration-dependent manner (Tables 3 and 4). Furthermore, the concentrations that inhibited COX-2 had no effect on COX-1. The decrease in PGE2 production after ACEO treatment corresponded with the decrease in COX activity in vitro (COX-1 and COX-2), particularly COX-2. EOs of *Illicium anisatum* constituted mainly of 1,8-cineole and demonstrated its ability for inhibiting NO and PGE2 production in LPS-stimulated RAW 264.7 cells, along with the decrease in iNOS and COX-2 expression [58]. Further, Beer et al. [59] have studied the inhibitory effect of 1,8-cineole on COX 1 and 2 activity and declared that 1,8 cineole is a potent and selective COX 2 blocker. Abundance of 1,8-cineole in ACEOs makes it an important candidate for its inhibitory activity.

### 3.8. Inhibition of Heat-Induced Hemolysis by ACEOs

Reports on the mechanism of action of NSAIDs on anti-inflammatory reaction are suggested to be exerted by stabilization of lysosomal membranes, which suppress the release of tissue-destroying enzymes which have been implemented in the pathogenesis of rheumatoid arthritis [60]. Protection against hypotonicity or heat-induced hemolysis by the red blood cell (RBC) membrane system is widely employed in the testing of drugs for anti-inflammatory activity [61]. Table 2 shows the percentage inhibition of RBC membrane lysis by the treatment of ACEOs. EO of leaf exhibited 83.32 ± 0.34% inhibition of RBC membrane lysis in closer rate to the dexamethasone (91.77 ± 0.56), commonly used drug in the autoimmune hemolytic anaemia [62] at 5 *μ*M.

### 3.9. Topical Anti-Inflammatory Activity

#### 3.9.1. Effects of ACEOs on TPA-Induced Skin Inflammation

**Figure 6:**
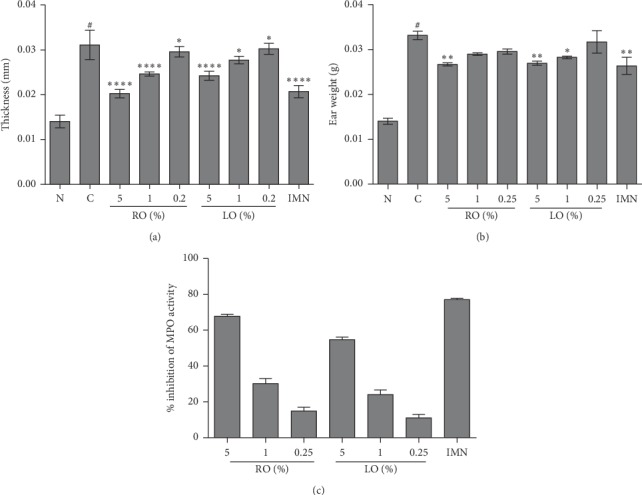
Effects of ACEOs on TPA-induced ear edema. Inhibition of TPA-induced ear edema by topical application of EO (5, 1, and 0.25%) was analyzed by measuring changes in ear thickness in millimeters (a), changes in ear weight in grams (b), and percentage inhibition of myeloperoxidase activity in tissue homogenates (c). N: normal; C: TPA-treated control group; RO: rhizome essential oil; LO: leaf essential oil; IMN: indomethacin at 0.5 mg/ear. Symbols “^*∗*^,” “^^*∗∗*^^,” “^^*∗∗∗*^^,” and “^^*∗∗∗∗*^^” represent a significant difference *p* < 0.05, *p* < 0.01, *p* < 0.001, and *p* < 0.0001 between TPA treatment and EO-treated animals. Symbol “^#^” represents a significant difference *p* < 0.05 between normal and TPA-treated groups.

**Figure 7:**
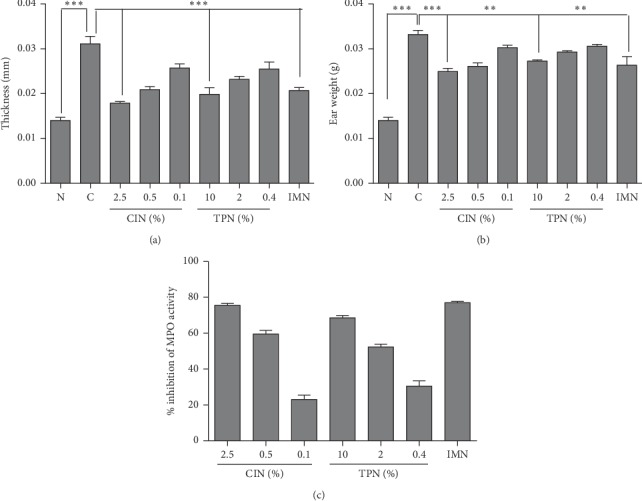
Effects of CIN and TPN on TPA-induced ear edema. Inhibition of TPA-induced ear edema by topical application of CIN (2.5, 0.5, and 0.1%) and TPN (10, 2, and 0.4%) was analyzed by measuring changes in ear thickness in millimeters (a), changes in ear weight in grams (b), and percentage inhibition of myeloperoxidase activity in tissue homogenates (c). N: normal; C: TPA-treated control group; CIN: 1,8-cineole; TPN: *α*-terpineol; IMN: indomethacin at 0.5 mg/ear. Symbols “^^*∗∗*^^” and “^*∗∗∗*^” represent a significant difference *p* < 0.01 and *p* < 0.001 between TPA treatment and compound-treated animals.

**Figure 8:**
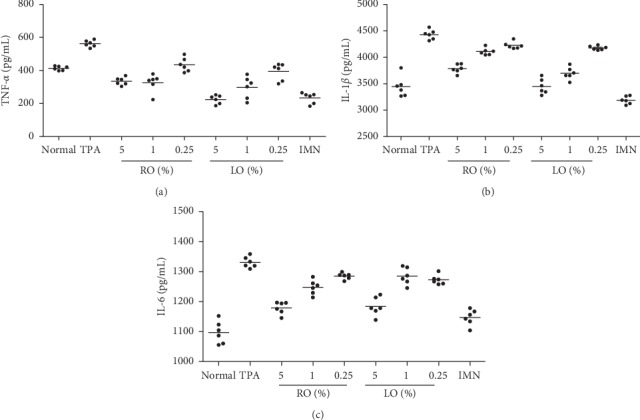
Effects of ACEOs on TPA-induced cytokine production. IL-1*β* levels in homogenate (a), IL-6 levels in homogenate (b), and TNF-*α* levels in homogenate (c). N: normal; TPA: TPA-treated control group; RO: rhizome essential oil (5, 1, and 0.25%); LO: leaf essential oil (5, 1, and 0.25%); IMN: indomethacin at 0.5 mg/ear. The bars represent the mean ± SEM (*n* = 6). All groups showed, *p* < 0.01, significantly reduced cytokine level than the control group.

**Figure 9:**
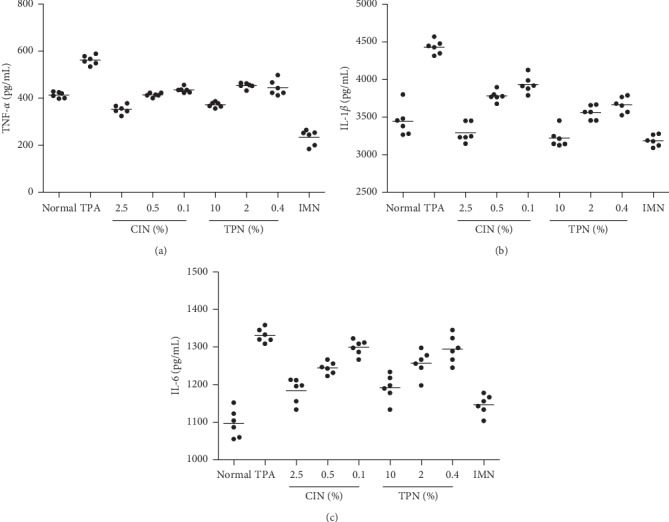
Effects of ACEOs on TPA-induced cytokine production. TNF-*α* levels in homogenate (a), IL-1*β* levels in homogenate (b), and IL-6 levels in homogenate (c). N: normal; TPA: TPA-treated control group; RO: rhizome essential oil; LO: leaf essential oil (5, 1, and 0.25%); IMN: indomethacin at 0.5 mg/ear. The bars represent the mean ± SEM (*n* = 6). All groups showed, *p* < 0.01, significantly reduced cytokine level than the control group.

Local application of TPA induces cutaneous inflammation and epidermal hyperplasia. Further, it stimulates infiltration of inflammatory cells, which releases large amounts of inflammatory mediators such as PGE2 and cytokines (TNF-*α*, IL-1*β*, and IL-6) [63]. In the current study, the therapeutic effect of ACEOs and its main constituents on TPA-induced skin inflammation was examined in a dose-dependent manner. Ear thickness was measured prior to the application of TPA. The concentrations of ACEO and compounds for testing were determined by doing a skin irritability test (data not shown). ACEO, CIN, and TPN presented significantly similar and effective anti-inflammatory activity in the experimental animal model used, which induced a strong dose-dependent edema inhibition (Figures 6(a) and 6(b) and Figures 7(a) and 7(b)). In particular, CIN showed a strong and similar edema inhibitory activity (29%) at 2.5% with indomethacin (25%) at 0.5 mg/ear. The mouse ear weight was reduced by 25% after indomethacin treatment, similar to rhizome EO (25%) and leaf EO (24.2%). TPN induced 23.4% edema reduction at 10% and 17% at 2% concentration. In summary, CIN and TPN which are the major compounds of AC presented a potent anti-inflammatory activity. To the best of our knowledge, it is the first report to demonstrate the topical anti-inflammatory activity of ACEO and also the compound TPN. Mounting evidences of the role of neutrophil enzyme, myeloperoxidase, in promoting oxidative stress in inflammatory pathologies and in many chronic inflammatory diseases such as atherosclerosis, glomerulonephritis, multiple sclerosis, rheumatoid arthritis, asthma, and cystic fibrosis makes it an important biochemical marker and a therapeutic target in antirheumatic drug development [64]. TPA-induced ear tissue homogenates showed high levels of MPO activity, and this activity was significantly reduced by the treatment with serial doses of ACEOs and compounds (Figures 6(c) and 7(c)). Further, the topical application also reduced the TNF-*α*, IL-1*β*, and IL-6 levels significantly in comparison with the TPA-induced group (Figures 8(a)–8(c)). In all the cases, rhizome oil exhibited more potential than the leaf oil (*p* < 0.05). Suggestive evidences on 1,8-cineole- and monoterpene-rich EOs on reducing airway inflammation, MPO activity, and decreased cytokine levels portrait the importance and efficacy of this topical study for therapeutic development [65]. Held et al. [28] have studied the oral anti-inflammatory effect of 1,8-cineole in various animal models, but the mechanism by which it exerts the activity was not clear. Our studies on the topical anti-inflammatory effect of CIN and TPN have explored the cytokine profile (Figures 9(a)–9(c)) and inhibition of MPO activity. From the results of this investigation, valuable research references can be provided for the clinical medicine or pharmaceutical application in the future.

#### 3.9.2. Histopathological Analysis of Mouse Ear Tissue

**Figure 10:**
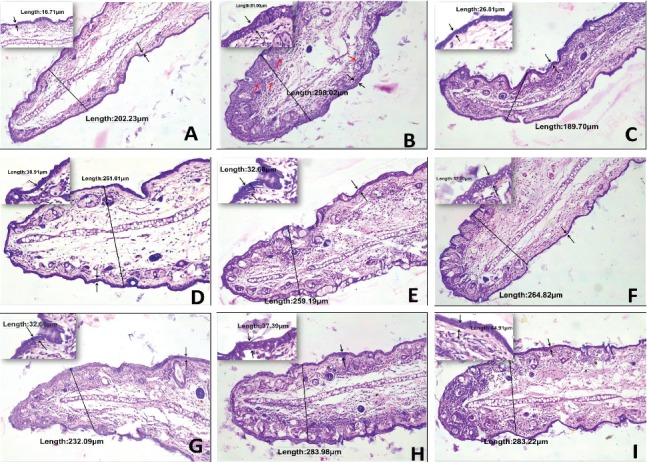
Histopathological analysis of TPA-induced ear edema in mice. (a) Normal mice ear, (b) TPA-treated mice, (c) indomethacin-treated mice, (d) rhizome oil-5%, (e) rhizome oil-1%, (f) rhizome oil-0.25%, (g) leaf oil-5%, (h) leaf oil-1%, and (i) leaf oil-0.25%. Images are the ear sections taken at 10x and 40x magnifications. The total thickness and epidermal area thickness were marked in micrometers, and the red arrows show the neutrophil infiltration in (b).

**Figure 11:**
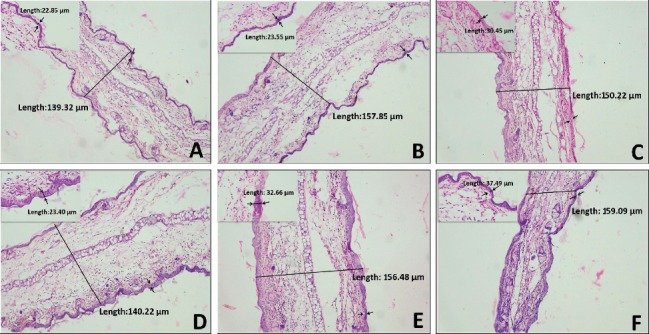
Histopathological analysis of TPA-induced ear edema in mice. (a) CIN-2.5%, (b) CIN-0.5%, (c) CIN-0.1%, (d) TPN-10%, (e) TPN-2%, and (f) TPN-0.4%. Images are the ear sections taken at 10x and 40x magnifications. The total thickness and epidermal area thickness were marked in micrometers, and the red arrows show the neutrophil infiltration in (b).

Further investigation on the H & E-stained ear biopsies from TPA-induced animals showed substantial increase in the ear thickness with vivid indication of edema, epidermal hyperplasia, and considerable neutrophil infiltration in the dermis associated with disruption in connective tissue. By comparison, 5% of ACEOs, CIN at 5%, and TPN at 10% treatment reduced ear thickness and affiliated pathological indicators to an extent comparable to the positive control, indomethacin, as shown in Figures 10 and 11. These results directly exemplify the effects of ACEO and major compounds in the amelioration of TPA-induced contact dermatitis.

#### 3.9.3. Effects of ACEOs on Formalin-Induced Pain and Edema

Acute and chronic inflammatory diseases are associated with neuralgic as well as inflammatory pain [66]. Since topical drug formulations of NSAIDs are amongst the most frequently administered over-the-counter (OTC) drugs, the introduction of a new preparation of this group requires any comparison of its anti-inflammatory and analgesic efficacy to the corresponding standard [67]. 2.5% formalin injection to the mice paw shows a biphasic response and exhibits behavioral pain by flinching and licking of the affected paw [68]. The present study was carried out to test the analgesic and antiedema effect of ACEOs in a dose-dependent manner. Results of the present study indicate distinctly the topical analgesic effect of ACEOs by suppressing the pain induced by inflammation in the second phase by significantly reducing the number of lickings and flinching (Table 5). EO of leaf showed significantly greater activity (66%) in reducing the number of flinches and lickings compared to the rhizome oil (52%) (*p* < 0.05). Analgesic efficacy of aromatic plants and EOs is attributed to the major constituents (Table 6), and the synergism between such chemical constituents is always the main concern of pharmaceutical industries in the interest of pain management [69].

## 4. Conclusion

The composition of essential oils collected was rich in oxygenated monoterpenes and its major compounds were 1, 8-cineole, *α*-Terpineol, and fenchyl acetate in rhizome, whereas leaf oil showcased the richness of camphor together with 1, 8-cineole and *α*-Terpineol. Further, ACEOs and main constituents ameliorate the production of inflammatory mediators such as nitric oxide, ROS, cytokines, and prostanoids *in vitro* and alleviate the edema and pain when applied topically in mice model of inflammation and pain. Further, ACEOs decrement the ontogeny of rat intestinal epithelial cells, human keratinocytes, and hepatocytes without affecting their viability. Hence, ACEOs featured the potential to be formulated into a more safe and sound alternative therapy for acute and chronic inflammatory maladies.

## Figures and Tables

**Table 1:** GC-MS profile of essential oils of *Alpinia calcarata* rhizome and leaves.

Compounds	Rhizome			Leaf		
	RT	RSI	Area	RT	RSI	Area
*Monoterpenes (M)*						
*α*-Pinene	5.29	926	0.07	5.32	900	0.06
*β*-Pinene	—	—	—	13.23	958	0.68
Ο-cymene	6.09	961	0.82	6.12	967	0.35
D-limonene	4.58	908	5.52	4.60	904	5.74
Pinocarvone	15.04	834	0.23	—	—	—
Myrtenal	17.15	909	0.65	17.17	860	0.04
γ-terpinene	5.51	929	0.39	5.54	917	0.59
*α*-Ocimene	5.66	929	0.26	5.68	933	0.70
Terpinolene	5.72	919	0.38	5.76	866	0.68
*α*-Copane	14.27	824	0.21	14.30	851	0.04
(+)-3-Carene	5.01	830	0.28	30.14	806	0.88
Fenchone	—	—	—	11.5	852	10
Pinocarveol	15.08	834	0.27	—	—	—
*α*-Terpinyl acetate	5.72	919	0.56	—	—	—
Total (M)			**9.64**			**19.76**

*Oxygenated monoterpenes (OM)*						
Eucalyptol (1,8-cineole)	4.76	948	38.45	4.76	954	31.08
*α*-Terpineol	19.03	838	11.62	19.95	881	10.31
Camphor	12.49	823	7.29	12.52	959	10.0
Bornyl acetate	15.57	933	0.38	15.59	930	0.51
Terpinen-4-ol	16.49	916	1.68	16.50	907	1.09
Fenchol	15.87	944	0.51	—	—	—
Fenchyl acetate	11.81	955	10.73	11.81	930	0.13
Linalool	18.28	863	0.62	18.32	866	0.39
Total (OM)			**71.28**			**53.51**

*Sesquiterpenes (S)*						
Carotol	30.50	923	3.36	30.50	924	6.53
Caryophyllene oxide	29.09	911	0.17	22.67	894	0.35
*α*-Bisabolene	33.99	831	0.05	18.51	886	0.61
*α*-Famesene	—	—	—	18.95	931	1.67
*β*-Sesquiphellandrene	34.95	826	—	34.59	826	0.25
α-Himachalene	—	—	—	35.34	893	0.55
*β*-Nerolidol	—	—	—	18.12	866	0.6
Cubedol	32.43	852	0.15	32.43	852	0.15
10-epi-Elemol	—	—	—	32.34	836	3.22
B-calarene	21.85	836	1.51	21.86	859	7.53
D-guaiene	20.28	916	0.14	—	—	—
Total (S)			**5.4**			**21.46**

*Aliphatic alcohol*						
2-Heptanol	7.31	918	0.11	7.35	899	0.12
6-epi-Shyobunol	31.18	802	1.13	28.57	797	1.82
Total			**1.24**			**1.94**

*Others*						
Phytol			—	40.98	899	0.88
Daucol	36.43	827	0.12	—	—	—
Trans-*α*-bergamotene	38.08	899	0.09	38.07	874	0.52
cis-p-Mentha-1 (7),8-dien-2-ol	24.82	846	0.07	19.60	792	0.14
2-Butanone, 4-phenyl-	—	—	—	6.71	841	0.45
2-Propenoic acid, 3-phenyl-, methyl ester	32.24	936	5.43	32.24	936	0.35
Methyl 9,11-octadecadienoate	37.75	826	—	19.47	797	0.51
Cholestan-3-ol, 2-methylene, (3*α*,5*α*)	36.93	830	0.1	35.55	816	
Octaethylene glycol monododecyl ether	43.48	788	0.12	39.59	755	
2-Methyl-4-(2,6,6-trimethylcyclohex-1-enyl)but-2-en-1-ol	14.61	808	0.48	37.38	811	0.27
5-Azulenemethanol						
1,2,3,3a,4,5,6,7-Octahydro-*α*,*α*,3,8-tetramethyl-, [3S-(3*α*,3a*β*,5*α*)]-	32.59	911	0.42	38.70	801	0.11
1H-benzocycloheptene,2,4a,5,6,7,8,9,9a-octahydro-3,5,5-trimethyl-9-methylene-	20.84	847	0.10	—	—	—
(-)-Myrtenol	23.21	921	0.23	23.22	872	0.07
Benzenepropanol, a-methyl-*β*-nitro-(R^*∗*^, R^*∗*^)-(.+-.)-	10.85	767	0.32	—	—	—
3,7-Cyclodecadiene-1-methanol, a,a,4,8-tetramethyl-, [s-(Z,Z)]	32.12	887	0.26	—	—	—
1-Heptatriacotanol	34.84	797	0.03	40.66	787	0.18
Cyclohexene-1-methyl-4-(1-methylethylidene)	4.24	944	0.08	4.26	932	0.11
Total			**7.85**			**2.7**
Overall total			**95.41**			**99.37**

**Table 2:** Intracellular ROS inhibition, superoxide anion scavenging, and inhibition of heat-induced hemolysis by ACEOs.

Treatment	Concentration (*μ*g/mL)	RLU ± SEM	Inhibition of intracellular ROS (%)^*∗∗*^	Superoxide inhibition (%)*∗*	Inhibition of heat-induced hemolysis (%)^*∗∗*^
Normal		298.53 ± 104.76			

Control		3587.56 ± 123.56^#^			

RO	50	568.9 ± 20.67^*∗∗*^	84.16 ± 5.56	13.45 ± 3.45	72.12 ± 0.25
	25	1305.76 ± 54.76^*∗∗*^	63.51 ± 7.34	10.54 ± 6.56	56.34 ± 0.45
	12.5	2135.98 ± 15.78^^*∗*^^	40.46 ± 5.87	8.45 ± 7.34	34.87 ± 0.76
	6.25	2582.45 ± 30.98^^*∗*^^	28.09 ± 6.34	6.44 ± 3.89	22.56 ± 1.22
	3.12	2902.23 ± 45.76^*∗∗*^	18.65 ± 4.34	2.56 ± 6.77	15.67 ± 2.34
	1.56	3276.67 ± 46.32^*∗∗*^	8.67 ± 6.56	2.31 ± 1.45	12.45 ± 0.23

LO	50	452.76 ± 67.23^*∗∗∗*^	87.32 ± 2.31	12.12 ± 3.24	83.32 ± 0.34
	25	1056.34 ± 59.56^*∗∗*^	70.56 ± 1.12	12.34 ± 1.11	67.23 ± 0.76
	12.5	1809.67 ± 23.34^*∗∗*^	49.56 ± 2.45	2.46 ± 6.57	54.09 ± 2.23
	6.25	2598.43 ± 67.23^*∗*^	27.57 ± 4.56	2.11 ± 1.35	24.76 ± 0.34
	3.12	2765.34 ± 25.67^*∗∗*^	22.91 ± 2.23	2.56 ± 2.23	14.65 ± 0.23
	1.56	3015.56 ± 34.87^*∗∗*^	15.98 ± 3.45	2.61 ± 2.45	10.23 ± 0.45

Dexamethasone	5 *µ*M	777.45 ± 34.56^*∗*^	78.33 ± 2.56	2.56 ± 1.77	91.77 ± 0.56

Data are mean ± SEM (*n* = 6). ^#^*p* < 0.05, significantly different from the normal group; ^*∗*^*p* < 0.05, ^*∗∗*^*p* < 0.01, and ^*∗∗∗*^*p* < 0.005, significantly different from the control group. RO: rhizome EO; LO: leaf EO.

**Table 3:** Effect of ACEOs on COX-1 enzyme activity.

Class	PGF_2*α*_(pg/mL)	Percentage of inhibition
Background tube	2.25 ± 0.75	
COX-1 100% initial activity tube	221 ± 14.5	

*Rhizome EO*		
0.5 *μ*g/mL	178 ± 4.5	19.45
5 *μ*g/mL	158 ± 2.23	28.5
50 *μ*g/mL	52 ± 5.23	76.47

*Leaf EO*		
0.5 *μ*g/mL	198 ± 5.45	10.4
5 *μ*g/mL	161 ± 2.56	27.14
50 *μ*g/mL	76 ± 2.12	65.61

*Indomethacin*		
10 *μ*g/mL	22 ± 3.45	90

**Table 4:** Effect of ACEOs on COX-2 enzyme activity.

Class	PGF_2*α*_ (pg/mL)	Percentage of inhibition (%)
*Background tube*	0.75 ± 0.1	
COX-2 100% initial activity tube	154 ± 4.5	

*Rhizome EO*		
0.5 *μ*g/mL	121 ± 4.5	21.4
5 *μ*g/mL	98 ± 1.23	36.3
50 *μ*g/mL	22 ± 2.56	85.7

*Leaf EO*		
0.5 *μ*g/mL	145 ± 2.78	5.84
5 *μ*g/mL	111 ± 1.54	27.92
50 *μ*g/mL	46 ± 1.12	70.12

NS-398	4 ± 2.21	97.4
10 *μ*mol/L		

**Table 5:** The effect of rhizome and leaf EOs on formalin-induced pain behavior and edema in mice at different concentrations.

Group	Phase I response		Phase II response		Paw volume (mL)	
	Flinching (freq.)	Licking (sec.)	Flinching (freq.)	Licking (sec.)	Baseline	4 h after injection
Normal	2 ± 2	0	2 ± 2	0	0.16 ± 0.2	—
Formalin-2.5%	20 ± 3.45^#^	72 ± 6.78^#^	112 ± 10.67^#^	225 ± 13.14^#^	0.18 ± 0.4	0.32 ± 0.02^*∗*^
RO-5%	6 ± 0.44	22 ± 3.24	35 ± 5.33	102 ± 5.67	0.16 ± 0.2	0.20 ± 0.02^*∗*^
RO-1%	10 ± 0.23	34 ± 2.56	54 ± 2.34	156 ± 3.45	0.18 ± 0.2	0.23 ± 0.03^*∗*^
RO-0.25%	18 ± 0.45	45 ± 1.24	82 ± 4.78	198 ± 4.35	0.16 ± 0.4	0.27 ± 0.02^*∗*^
LO-5%	3 ± 0.45	18 ± 2.45	28 ± 5.23	76 ± 5.67	0.17 ± 0.2	0.18 ± 0.02^*∗*^
LO-1%	8 ± 0.34	32 ± 2.23	36 ± 2.76	112 ± 6.89	0.16 ± 0.2	0.21 ± 0.04^*∗*^
LO-0.25%	14 ± 0.56	40 ± 3.23	62 ± 5.45	154 ± 4.78	0.18 ± 0.4	0.24 ± 0.02^*∗*^
Diclofenac-1%	2 ± 0.21	12 ± 1.24	22 ± 2.34	54 ± 1.34	0.16 ± 0.2	0.17 ± 0.02^*∗*^

Data are mean ± SE. The numbers are frequencies (freq.) of flinching and total time (seconds; sec.) spent licking the formalin-injected paw. The behavioral response was measured at a total of 30 min after subplantar injection. *p* < 0.05 compared with the formalin-treated group (*n* = 6), ^#^*p* < 0.05 compared with the normal group. Baseline values of paw volume represent preinjection diameters of paws. ^*∗*^*p* < 0.05, compared with the formalin-treated group (*n* = 6) (ANOVA with Tukey's test). RO: rhizome EO; LO: leaf EO.

**Table 6:** The effect of 1,8-cineole and *α*-terpineol on formalin-induced pain behavior and edema in mice at different concentrations.

Group	Phase I response		Phase II response		Paw volume (mL)	
	Flinching (freq.)	Licking (sec.)	Flinching (freq.)	Licking (sec.)	Baseline	4 h after injection
Normal	2 ± 2	0	2 ± 2	0	0.16 ± 0.2	—
Formalin-2.5%	20 ± 3.45^#^	72 ± 6.78^#^	112 ± 10.67^#^	225 ± 13.14^#^	0.18 ± 0.4	0.32 ± 0.02^^*∗*^^
CIN-2.5%	8 ± 0.42	12 ± 1.21	15 ± 1.22	87 ± 2.56	0.16 ± 0.2	0.22 ± 0.02^^*∗*^^
CIN-0.5%	16 ± 0.32	36 ± 1.56	24 ± 1.45	108 ± 2.45	0.17 ± 0.2	0.27 ± 0.02^^*∗*^^
CIN-0.1%	18 ± 0.56	45 ± 2.24	52 ± 3.46	178 ± 2.55	0.16 ± 0.2	0.29 ± 0.02^^*∗*^^
TPN-10%	13 ± 0.22	21 ± 2.56	18 ± 4.67	96 ± 6.78	0.17 ± 0.2	0.24 ± 0.02^^*∗*^^
TPN-2%	18 ± 0.12	35 ± 2.89	46 ± 3.56	122 ± 5.45	0.16 ± 0.2	0.21 ± 0.02^^*∗*^^
TPN-0.4%	21 ± 0.14	49 ± 1.23	72 ± 1.34	164 ± 7.4	0.16 ± 0.4	0.29 ± 0.02^^*∗*^^
Diclofenac-1%	2 ± 0.12	12 ± 3.24	22 ± 4.56	54 ± 3.23	0.16 ± 0.2	0.29 ± 0.02^^*∗*^^

Data are mean ± SEM. The numbers are frequencies (freq.) of flinching and total time (seconds; sec.) spent licking the formalin-injected paw. The behavioral response was measured at a total of 30 min after subplantar injection. *p* < 0.05 compared with the formalin-treated group (*n* = 6), ^#^*p* < 0.05 compared with the normal group. Baseline values of paw volume represent preinjection diameters of paws. ^*∗*^*p* < 0.05, compared with the formalin-treated group (*n* = 6) (ANOVA with Tukey's test). CIN: 1,8-cineole; TPN: *α*-terpineol.

## Data Availability

The authors confirm that the data supporting the findings of this study are available within the article.

## Abbreviations

AC: *Alpinia calcarata*
EO: Essential oil
ACEOs: *Alpinia calcarata* essential oils
RO: Rhizome oil
LO: Leaf oil.

## Supplementary Materials

Supplementary Materials Graphical abstract.
