# Supplementary figures and images for: Topical Anti-Inflammatory Activity of Essential Oils of Alpinia calcarata Rosc., Its Main Constituents, and Possible Mechanism of Action
Source: Evid Based Complement Alternat Med. 2020 Apr 27;2020:2035671. doi: 10.1155/2020/2035671 (PMC7204338; doi:10.1155/2020/2035671)

## Graphical abstract:

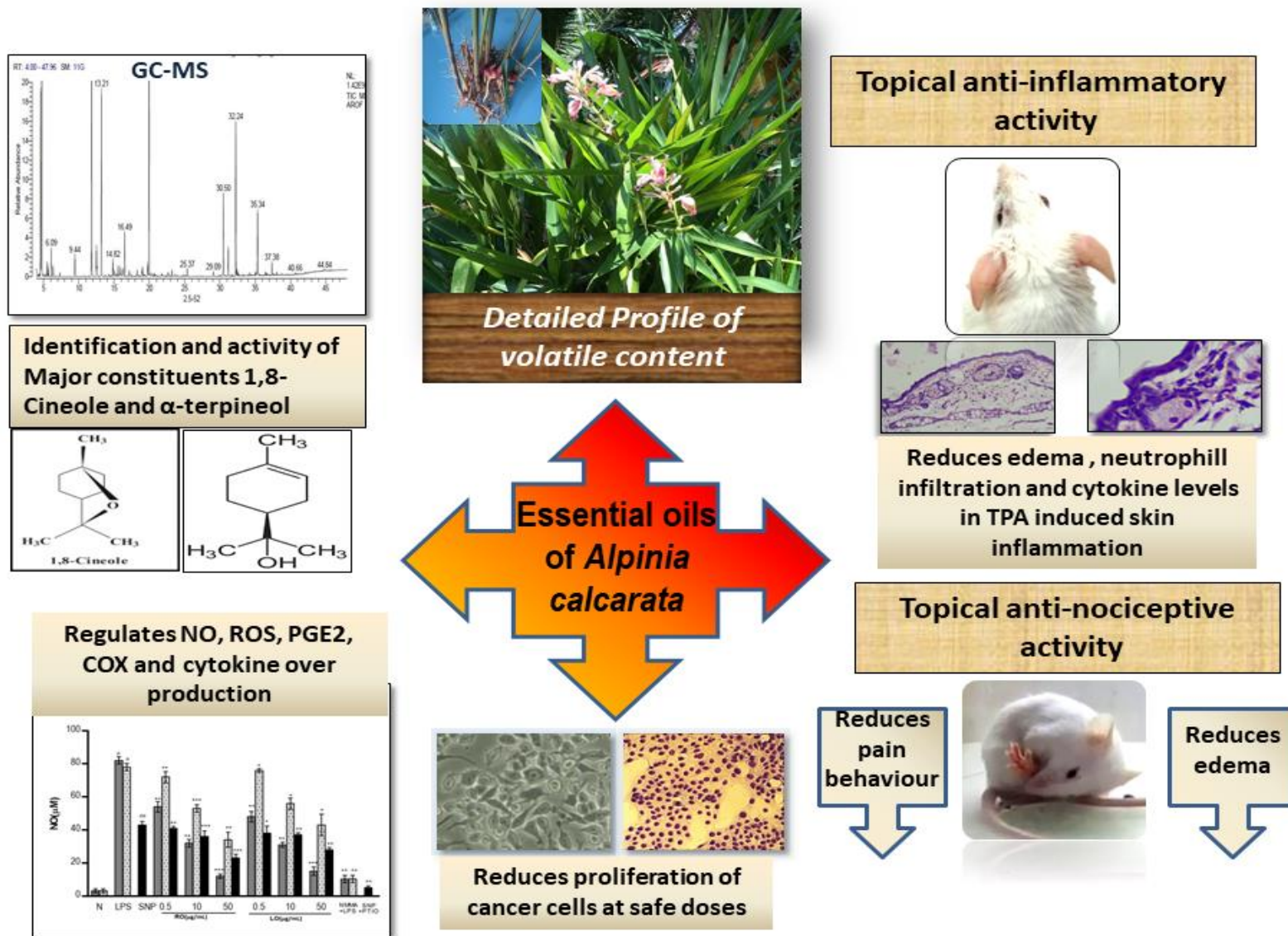

Supplement: Supplementary Materials — Graphical abstract. [file 2035671.f1.pdf]
